# Experiences and perceptions of weight change among people living with HIV receiving antiretroviral therapy: a qualitative systematic review and meta-synthesis

**DOI:** 10.3389/fpubh.2026.1947513

**Published:** 2026-10-27

**Authors:** Shuhao Zhang, Jiahao Gao, Yin Liu, Xuesong Jiang

**Affiliations:** 1 School of Nursing (Nursing School of Smart Healthcare Industry), Henan University of Chinese Medicine, Zhengzhou, Henan, China; 2 Department of Infection Control, The First Affiliated Hospital of Henan University of Chinese Medicine, Zhengzhou, Henan, China

**Keywords:** antiretroviral therapy, HIV infection, meta-synthesis, qualitative research, weight change, weight management

## Abstract

**Background:**

With improved survival following the widespread use of antiretroviral therapy (ART), weight change has become an important concern in the long-term care of people living with HIV. Weight loss and gain may carry medical, psychological, and sociocultural meanings and may influence body image, treatment perceptions, ART adherence, and weight-management behaviors. This review synthesized qualitative evidence on experiences and perceptions of weight change among people living with HIV receiving ART.

**Methods:**

A systematic search of PubMed, Embase, Web of Science, Scopus, CINAHL, ProQuest, PsycINFO, China National Knowledge Infrastructure, Wanfang Data, VIP Database, and the Chinese Biomedical Literature Service System was conducted from database inception to June 2026 to identify qualitative studies examining experiences of weight change among people living with HIV receiving antiretroviral therapy. Methodological quality was assessed using the Joanna Briggs Institute Critical Appraisal Checklist for Qualitative Research. Findings were synthesized using JBI meta-aggregation, and confidence was evaluated using CERQual.

**Results:**

Seven studies were ultimately included, from which 57 findings were extracted, grouped into 12 categories, and synthesized into three overarching findings: the multiple meanings of weight change, the attributions and impacts of weight change, and the barriers to and support for weight management. Confidence was high for the first finding and moderate for the other two.

**Conclusion:**

Interpretations of weight change among people living with HIV are shaped by prior illness experiences, beliefs about antiretroviral therapy, and prevailing sociocultural norms, and may, in turn, affect psychosocial well-being, physical health, and treatment-related behaviors. HIV care should integrate routine monitoring of weight and metabolic risk with sustained, individualized support. Because this synthesis captures participants’ perceptions and causal attributions, it does not establish that ART, any individual antiretroviral agent, or a specific regimen caused the reported weight changes.

**Systematic review registration:**

PROSPERO, CRD420261425511.

## 1 Introduction

HIV remains a major global public health challenge. In 2025, an estimated 40.9 million people were living with HIV worldwide, of whom approximately 32.1 million were receiving antiretroviral therapy (ART) (1). ART has markedly improved survival and, for many people, made HIV a manageable chronic condition requiring lifelong care (2). At the same time, the clinical meaning of weight change has shifted. Before effective ART became widely available, weight loss and wasting often indicated disease progression and impaired immune function; in the current treatment era, weight recovery after ART initiation, excessive weight gain, and obesity have become increasingly important concerns (3). These weight trajectories are shaped by disease status, recovery following treatment initiation, individual and environmental factors, and ART regimen characteristics, rather than by any single antiretroviral drug class (4). Among these changes, excessive weight gain has attracted particular attention. Approximately 13% of people initiating ART gain more than 10% of their baseline body weight within the first year (5), and excessive post-treatment weight gain has been associated with diabetes, metabolic syndrome, and cardiometabolic events (6, 7). Weight change also has important psychosocial implications. Changes in weight and appearance may disrupt body image and have been associated with depression, anxiety, social withdrawal, and other forms of psychological distress (8). Weight is therefore not merely a clinical measure requiring surveillance; it may also influence how people interpret their illness and treatment and how they engage in self-management. Current guidelines accordingly recommend incorporating weight monitoring and weight-management counseling into comprehensive HIV care (9). Although qualitative studies have described these experiences in different settings, their findings remain fragmented across contexts. This review therefore used a meta-aggregation approach to synthesize experiences of weight change among people living with HIV in the context of ART. It aimed to clarify how participants perceived weight change and how it affected their physical and psychological health and treatment-related behaviors. The review also sought to identify barriers to weight management and unmet support needs, thereby informing patient-centered weight assessment, risk communication, and individualized care. Accordingly, the review question was: How do people living with HIV who are receiving or have previously received ART experience and interpret weight change and weight-management practices in the context of treatment, follow-up, and everyday life?

## 2 Materials and methods

### 2.1 Study design and reporting

This qualitative systematic review and meta-synthesis was conducted using the JBI meta-aggregation approach and reported in accordance with the Preferred Reporting Items for Systematic Reviews and Meta-Analyses (PRISMA) 2020 statement (10). The review question was structured using the PICo framework to reflect the experiential focus of qualitative evidence synthesis, with Population, Phenomenon of Interest, and Context specified *a priori*. This systematic review was registered in PROSPERO (registration number: CRD420261425511; https://www.crd.york.ac.uk/PROSPERO/view/CRD420261425511).

### 2.2 Search strategy

A comprehensive search was conducted across PubMed, Embase, Web of Science, Scopus, CINAHL, ProQuest, PsycINFO, China National Knowledge Infrastructure, Wanfang Data, VIP Database, and the Chinese Biomedical Literature Service System from inception through June 22, 2026. Controlled vocabulary and free-text terms were adapted to each database and organized around four concepts: HIV, antiretroviral therapy, weight change, and qualitative research. Terms within each concept were combined using “OR” and the four concept groups were linked using “AND.” Equivalent Chinese terms were used for the Chinese databases. The reference lists of the included studies and other relevant publications were also screened to identify additional records. Complete search strategies for all databases are provided in Supplementary File 1.

### 2.3 Inclusion and exclusion criteria

Using the PICo framework, the Population comprised people living with HIV who were currently receiving or had previously received ART; the Phenomenon of Interest comprised experiences and perceptions of weight gain, weight loss, other weight changes, and related weight-management practices after ART initiation; and the Context comprised treatment, regimen switching, clinical follow-up, and everyday-life settings. Eligible studies met all of the following criteria: (1) participants were people living with HIV who were currently receiving or had previously received ART; (2) the study explored experiences or perceptions of weight gain, weight loss, other weight changes, or related weight-management practices; (3) these experiences occurred after ART initiation, including during ongoing treatment, regimen switching, clinical follow-up, or everyday life; and (4) the study used a primary qualitative design or a mixed-methods design with a separately extractable qualitative component. For studies involving multiple stakeholder groups, only findings relating to people living with HIV were included when these data could be clearly distinguished from those of other participants. Studies were excluded if they were: (1) reviews, conference abstracts, theses, or dissertations; (2) published in languages other than Chinese or English; (3) mixed-methods studies without a separately extractable qualitative component; (4) duplicate reports of the same study; or (5) reports for which the full text could not be obtained.

### 2.4 Study selection and data extraction

All retrieved records were imported into EndNote 21, and duplicate records were removed. Two reviewers independently screened the titles and abstracts and subsequently assessed potentially eligible full-text reports against the eligibility criteria. Disagreements were resolved through discussion and, when necessary, adjudication by a third reviewer. Two reviewers independently extracted data using a structured data extraction form. The extracted information included the first author, year, country, design and data collection, participants, ART treatment, focus of interest, and key findings. Discrepancies in data extraction were resolved through discussion and, when necessary, adjudication by a third reviewer.

### 2.5 Methodological quality assessment

Two reviewers independently appraised the methodological quality of the included studies using the 2016 version of the Joanna Briggs Institute Critical Appraisal Checklist for Qualitative Research (11). The checklist comprises 10 items rated as “yes,” “no,” “unclear,” or “not applicable.” Based on prespecified criteria, studies were categorized as grade A when all appraisal criteria were met and methodological limitations were minimal, grade B when some criteria were unmet or unclear and methodological limitations were moderate, and grade C when a substantial number of criteria were unmet and methodological limitations were serious. Only studies rated as grade A or B were included in the synthesis. Disagreements were resolved through discussion, with a third reviewer consulted when necessary.

### 2.6 Data synthesis

Study findings were synthesized using the JBI meta-aggregation approach and corresponding methodological guidance for qualitative evidence synthesis (12). Two reviewers read the included studies several times and independently extracted unequivocal findings supported by participant quotations or interpretations reported by the original authors. Each finding was assigned a unique code based on the first author’s name and its order of extraction. Findings with similar meanings were grouped into categories while preserving their original context, and related categories were subsequently integrated into synthesized findings. Disagreements regarding the extraction, categorization, or synthesis of findings were resolved through discussion and, when necessary, consultation with a third reviewer.

### 2.7 Assessment of confidence in the evidence

Confidence in each synthesized finding was assessed using the Confidence in the Evidence from Reviews of Qualitative Research (CERQual) approach (13). The assessment considered four domains: methodological limitations, relevance, coherence, and adequacy of data. Each synthesized finding was initially assigned high confidence and downgraded when concerns were identified in one or more domains, yielding an overall confidence rating of high, moderate, low, or very low. Two reviewers independently assessed each synthesized finding and compared their judgments. Disagreements were resolved through discussion and, when necessary, consultation with a third reviewer.

## 3 Results

### 3.1 Study selection results

The database searches yielded 2,300 records, and two additional records were identified by screening the reference lists. After 1,156 duplicate records were removed, 1,146 records underwent title and abstract screening. Of these, 67 reports were assessed in full. Seven studies met the eligibility criteria and were included in the meta-synthesis (14–20). The study selection process and reasons for excluding reports after full text assessment are presented in Figure 1.

**Figure 1:**
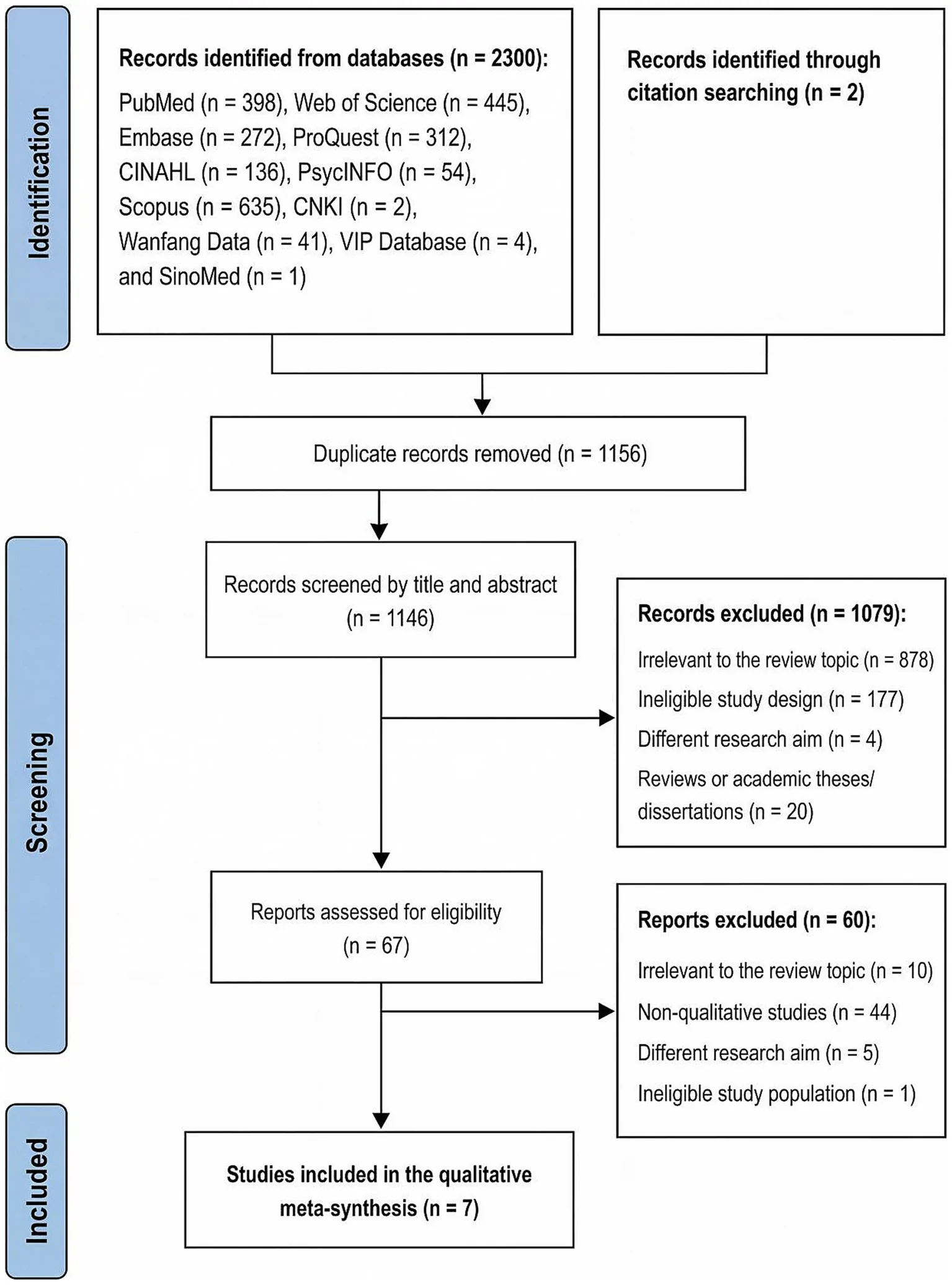
PRISMA flow diagram of the study selection process.

### 3.2 Characteristics and quality assessment of the included studies

The characteristics of the included studies are presented in Table 1. The included evidence was geographically concentrated: five studies were conducted in sub-Saharan Africa and two in the United States. Several studies recruited selected clinical subgroups, including women receiving dolutegravir (DTG), participants with obesity, and participants with obesity and/or type 2 diabetes mellitus, which may have increased the representation of people with pre-existing or clinically salient weight concerns. Methodological quality assessment classified one of the seven studies as grade A and the remaining six as grade B, as shown in Table 2.

**Table 1:** Characteristics of the included studies.

Authors	Year	Country	Design and data collection	Participants and clinical characteristics	ART characteristics	Focus of interest	Key findings
Manyaapelo et al. (14)	2026	South Africa	Qualitative study; semi-structured interviews	26 PLWH with obesity Age: NR; sex: NR; BMI/weight status: BMI ≥ 30 kg/m^2^ (obesity); major comorbidities: NR.	Received EFV-based ART for ≥6 months, followed by switching to a TLD regimen ART-naïve/experienced status: ART-experienced; regimen: EFV-based ART followed by TLD; duration: EFV-based ART ≥6 months; post-switch duration NR.	Perceptions of weight gain after switching to a TLD regimen and preferences for interventions among PLWH	Five themes: body size preference; preference for smaller body size; large body size and ill health; weight gain and ART; weight loss intervention preference.
Suvada et al. (15)	2025	Kenya	Explanatory sequential mixed-methods study; semi-structured interviews; rapid qualitative analysis	24 PLWH Interview subset: mean age 47.4 years (SD 10.1); sex distribution NR for the interview subset; mean BMI 27.7 kg/m^2^ (SD 8.6): underweight *n* = 3, normal *n* = 7, overweight *n* = 9, obesity *n* = 5; major comorbidity distribution NR for the interview subset.	Received ART for ≥6 months; regimen was not reported ART-naïve/experienced status: ART-experienced; ART duration ≥6 months; regimen NR.	Perceptions of weight change, weight management, and noncommunicable disease risk among PLWH	Five themes: perceptions of NCD causes and risk; awareness and perceptions of body size associations with health and individual characteristics; experiences with weight loss and gain; experiences of weight-related stigma and weight-related discrimination; experiences with service delivery for NCDs.
Koffi et al. (16)	2025	Côte d’Ivoire	Descriptive qualitative study; semi-structured interviews; thematic analysis	15 PLWH with obesity and/or type 2 diabetes mellitus Age 47–62 years (median 57); sex: 9 women/6 men; metabolic conditions: diabetes only *n* = 7, obesity only *n* = 6, both diabetes and obesity *n* = 2; individual BMI values NR.	Received ART; regimen and treatment duration were not reported ART-naïve/experienced breakdown: NR; regimen and ART duration NR.	Perceptions of obesity and experiences of dietary and physical activity management among PLWH	Three themes: diabetes in people living with HIV: between worry and anxiety; obesity: a socially accepted comorbidity among people living with HIV; barriers to physical activity and healthy diet to manage diabetes and obesity.
Kaufman et al. (17)	2025	United States	Qualitative study; semi-structured interviews; inductive thematic analysis	61 PLWH Age 23–65 years; gender: 51% cisgender men, 36% cisgender women, 13% transgender/nonbinary; baseline weight/BMI: NR; major comorbidities: NR.	Previously or currently receiving ART; regimen and treatment duration were not reported ART exposure: participants were currently or previously receiving ART; regimen and treatment duration NR.	Attributions and experiences of weight change after ART initiation or regimen switching, and related health behaviors among PLWH	Four themes: weight changes were attributed to external and internal factors; weight management was seen as an individual responsibility; feeling in control of one’s health was linked to body image satisfaction; one’s sense of control related to health behaviors in divergent ways.
Dube et al. (18)	2025	South Africa	Exploratory qualitative substudy; focus group discussions; inductive thematic analysis	Five focus groups of PLWH, with 7–12 participants per group Age: adults; exact age distribution NR; sex composition: two male-only, two female-only, and one mixed-gender PLWH focus group; baseline weight/BMI and major comorbidities: NR.	Received stavudine-based ART; treatment duration was not reported ART-naïve/experienced status: ART-experienced; regimen: stavudine-based ART; treatment duration NR.	Perceptions of weight change among PLWH and its influence on reporting weight loss during tuberculosis screening	Three themes: perceptions and attitudes toward weight change; ideal body size and shape; negative impact of weight loss on participant’s daily life.
Alhassan et al. (19)	2022	Uganda	Descriptive qualitative study; semi-structured interviews; thematic analysis	25 women living with HIV Age: 18–24 years *n* = 10, 25–49 years *n* = 10, >49 years *n* = 5; sex: all women; baseline BMI/weight status NR; diabetes was reported by 2 participants.	Received DTG-based ART for ≥3 months Participants had initiated or transitioned to DTG-based ART; exact ART-naïve versus ART-experienced breakdown NR; DTG exposure ≥3 months.	Perceptions of weight gain after initiating a DTG-based regimen and its effects on body image and treatment adherence among women living with HIV	Eight themes: weight-gain perceived association with DTG-based regimen; WLHIV aversion to thinness; body size preference; concerns about body weight; weight gain was underreported; effect of weight change on medication adherence; gaps in risk communication; barriers to monitoring and treating weight gain and related complications.
Abel et al. (20)	2018	United States	Qualitative study; semi-structured interviews; grounded theory analysis	22 PLWH Median age 44.5 years (IQR 30.5–52.0); sex: 14 men/8 women; BMI: normal *n* = 7, overweight *n* = 6, obesity *n* = 9; median BMI 26.9 kg/m^2^ in men and 30.5 kg/m^2^ in women; diabetes *n* = 6.	Received ART; regimen and treatment duration were not reported ART-naïve/experienced status: ART-experienced/receiving ART; regimen and treatment duration NR.	Perceptions of weight change and body image, and experiences of lifestyle-based weight management among PLWH	Eleven themes: dominant food culture; food and family; obesity as community norm; childhood food access; knowledge base; work environment; cost of healthy food; lack of time; Thin = ill; weight as a buffer against future illness; weight hides HIV-related body changes.

ART, antiretroviral therapy; DTG, dolutegravir; EFV, efavirenz; NCD, noncommunicable disease; PLWH, people living with HIV; TLD, tenofovir disoproxil fumarate/lamivudine/dolutegravir. NR, not reported in the primary publication.

**Table 2:** Quality evaluation results of included studies (*n* = 7).

Included study	①	②	③	④	⑤	⑥	⑦	⑧	⑨	⑩	Quality level
Manyaapelo et al. (14)	U	Y	Y	Y	Y	U	U	Y	Y	Y	B
Suvada et al. (15)	U	Y	Y	Y	Y	Y	U	Y	Y	Y	B
Koffi et al. (16)	U	Y	Y	Y	Y	U	U	Y	Y	Y	B
Kaufman et al. (17)	Y	Y	Y	Y	Y	Y	Y	Y	Y	Y	A
Dube et al. (18)	U	Y	Y	Y	Y	U	U	Y	Y	Y	B
Alhassan et al. (19)	Y	Y	Y	Y	Y	Y	U	Y	Y	Y	B
Abel et al. (20)	U	Y	Y	Y	Y	U	Y	Y	Y	Y	B

Y, yes; N, no; U, unclear. ① Congruity between the stated philosophical perspective and the research methodology; ② congruity between the research methodology and the research question or objectives; ③ congruity between the research methodology and the methods used to collect data; ④ congruity between the research methodology and the representation and analysis of data; ⑤ congruity between the research methodology and the interpretation of results; ⑥ a statement locating the researcher culturally or theoretically; ⑦ the influence of the researcher on the research, and vice versa, is addressed; ⑧ participants and their voices are adequately represented; ⑨ the study is ethical according to current criteria or has evidence of ethical approval; ⑩ the conclusions drawn in the research report flow from the analysis or interpretation of the data.

### 3.3 Meta-synthesis results

A total of 57 unequivocal findings were extracted from the seven studies, grouped into 12 categories, and further synthesized into three findings. The CERQual assessment of confidence in the synthesized findings is presented in Table 3. In this review, “weight change” is used as an umbrella term encompassing several distinct phenomena represented in the primary qualitative studies: illness-associated or unintentional weight loss; weight restoration after ART initiation that may reflect a return-to-health process; persistent, excessive, or unwanted weight gain; obesity or body-size concerns; and experiences of trying to manage weight. These phenomena were synthesized because their experiential meanings overlapped, but they should not be interpreted as clinically equivalent. To improve readability, representative participant quotations are summarized in Table 4; the main text focuses on the synthesized meaning of each category, with only selected brief quotations retained where they clarify meaning.

**Table 3:** CERQual assessment of confidence in the synthesized findings on weight-change experiences among people living with HIV receiving antiretroviral therapy.

Synthesized finding	Number of contributing studies	CERQual confidence rating	Explanation
The multiple meanings of weight change	7 (14–20)	High	The contributing studies had some methodological limitations, mainly related to researcher reflexivity. Nevertheless, the finding was directly relevant to the review question, coherent across all seven studies, and supported by rich and adequate data from five countries.
The attributions and impacts of weight change	6 (14–19)	Moderate	The contributing studies had some methodological limitations, but the evidence was generally relevant to the review question and coherent across studies. Data were reasonably adequate overall, although support was uneven across the different components of the finding, with some components represented by fewer and less detailed data. The small and thematically heterogeneous evidence base and uneven contribution across components further limit the breadth of inference.
The barriers to and support for weight management	6 (14–17, 19, 20)	Moderate	The contributing studies had some methodological limitations, while the evidence was generally relevant and coherent across settings. Data on barriers to weight management were reasonably adequate, but evidence concerning specific support needs was less extensive, resulting in moderate confidence in the overall finding. Evidence for specific support needs was contributed by fewer and less detailed data than evidence concerning barriers.

**Table 4:** Representative participant quotations supporting the synthesized categories.

Synthesized finding	Category	Representative quotation	Source study
The multiple meanings of weight change	Category 1: Diseases and treatment indications	“When you are thin, you are not on treatment.”	Manyaapelo et al. (14)
The multiple meanings of weight change	Category 2: Inadvertent disclosure of HIV status and social stigma	“Always when you are growing slim… person thinks of HIV.”	Suvada et al. (15)
The multiple meanings of weight change	Category 3: The positive meanings of weight gain	“You see as I am fat, I can feel that all is well with me.”	Manyaapelo et al. (14)
The multiple meanings of weight change	Category 4: Preference for a moderate or slightly above-average weight	“I like the medium size because you are not too heavy… and… people [do not] think you are sick.”	Suvada et al. (15)
The attributions and impacts of weight change	Category 5: Multifactorial attributions of weight change	“The treatment [ART] had contributed to my weight gain.”	Manyaapelo et al. (14)
The attributions and impacts of weight change	Category 6: Risks to physical health	“I fear that if I gained more weight I might get a heart attack.”	Manyaapelo et al. (14)
The attributions and impacts of weight change	Category 7: Psychological and body image impacts	“I do not think I’m attractive anymore, I do not think I’m worthy anymore.”	Kaufman et al. (17)
The attributions and impacts of weight change	Category 8: ART adherence and treatment decisions	“I will stop taking them since I do not see its results.”	Dube et al. (18)
The barriers to and support for weight management	Category 9: Personal responsibility and self-efficacy	“So I lost 20 pounds and that makes me feel good.”	Kaufman et al. (17)
The barriers to and support for weight management	Category 10: Weight management strategies	“I cannot [workout] by myself; it would be better I [exercise] with a group.”	Manyaapelo et al. (14)
The barriers to and support for weight management	Category 11: Family and socioeconomic barriers	“If you look at it now, it’s expensive to eat healthy.”	Abel et al. (20)
The barriers to and support for weight management	Category 12: Professional support and integrated care	“The services provided [need] to be under the same roof—HIV, blood pressure and diabetes.”	Suvada et al. (15)

#### 3.3.1 Synthesized finding 1: The multiple meanings of weight change

##### 3.3.1.1 Category 1: Diseases and treatment indications

Participants often interpreted weight loss as a sign of untreated or worsening HIV, poor treatment response, or comorbid disease such as tuberculosis; previous experiences of illness-related wasting intensified these concerns. Conversely, consistent ART use and maintenance of a healthier appearance were perceived as signs of treatment success and recovery (14, 15, 18, 20).

##### 3.3.1.2 Category 2: Inadvertent disclosure of HIV status and social stigma

In several sociocultural settings, thinness was perceived as a visible marker of HIV infection, creating concerns about unintended disclosure, gossip, derogatory labeling, social exclusion, and withdrawal from social contact. Low body weight could also be interpreted as evidence of severe illness, poverty, or hardship (15, 18, 19).

##### 3.3.1.3 Category 3: The positive meanings of weight gain

Weight gain was often assigned positive meanings. Participants linked a fuller body size to good health, recovery, successful treatment, attractiveness, strength, and socioeconomic well-being, and positive feedback from others could further reinforce favorable views of weight gain after prior illness-related wasting (14–19).

##### 3.3.1.4 Category 4: Preference for a moderate or slightly above-average weight

Participants generally did not express an unconditional preference for obesity. Rather, many preferred a moderate or slightly larger body size that avoided illness-associated thinness while preserving mobility; as one participant described, the preferred body size was a “medium size” rather than being too thin or too heavy (15). Previous severe wasting also led some participants to view additional body weight as a nutritional reserve against future illness (14, 15, 20).

#### 3.3.2 Synthesized finding 2: The attributions and impacts of weight change

##### 3.3.2.1 Category 5: Multifactorial attributions of weight change

Participants offered multiple explanations for weight change. Some attributed weight gain to ART, increased appetite, or medication-related effects, whereas others linked weight fluctuation to gastrointestinal symptoms, depression, emotional well-being, dietary intake, or overeating. These accounts represent participants’ own causal attributions and should not be interpreted as evidence that ART or a specific regimen caused the reported weight changes (14, 17).

##### 3.3.2.2 Category 6: Risks to physical health

Participants perceived higher body weight as increasing the risks of cardiovascular disease, diabetes, hypertension, high cholesterol, reduced mobility, and functional limitation. The threshold at which weight became concerning varied, and some participants regarded weight gain as problematic mainly when it substantially interfered with daily activities (14–16, 19).

##### 3.3.2.3 Category 7: Psychological and body image impacts

Both weight loss and unwanted substantial weight gain were associated with psychological distress, a reduced sense of control, body dissatisfaction, diminished self-worth, and altered social participation. Some participants also described cycles involving depression, emotional eating, further weight gain, and worsening body dissatisfaction (17–19).

##### 3.3.2.4 Category 8: ART adherence and treatment decisions

Participants reported that perceived medication-related weight loss or gain could influence confidence in treatment and intentions regarding ART, including considering treatment discontinuation or interruption. Stigma associated with visible weight loss could also contribute to missed doses, whereas perceived beneficial weight gain could reinforce adherence (17–19). These findings describe participants’ perceptions and treatment-related decisions; they do not establish that ART caused the weight changes.

#### 3.3.3 Synthesized finding 3: The barriers to and support for weight management

##### 3.3.3.1 Category 9: Personal responsibility and self-efficacy

Weight management was frequently framed as a personal responsibility involving diet, self-discipline, and physical activity. Successful weight loss or maintenance strengthened self-efficacy, whereas inability to achieve a desired weight caused frustration; normalization of obesity within families or communities could also contribute to fatalistic beliefs about weight-related risk (17, 20).

##### 3.3.3.2 Category 10: Weight management strategies

Participants used walking, exercise, dietary changes, and other lifestyle modifications to manage weight. Group-based activity could be more acceptable than exercising alone—one participant stated, “I cannot [workout] by myself” (14)—and successful behavior change could restore a sense of control. Acceptance of pharmacological weight-management options was conditional on side effects and treatment burden, while additional tablets, repeated clinic visits, and weekly injections could reduce acceptability. Physician recommendation and preparing food separately from family members could facilitate participation in weight-management efforts (14, 17, 19, 20).

##### 3.3.3.3 Category 11: Family and socioeconomic barriers

Financial pressures, household responsibilities, the high cost of healthier foods, family food norms, and ridicule of dietary restraint were important barriers to weight management. Long working hours, limited time for meal preparation, and the convenience of fast food further constrained sustained dietary change (16, 19, 20).

##### 3.3.3.4 Category 12: Professional support and integrated care

Participants described support from clinicians and nutrition professionals and expressed a preference for integrated HIV and noncommunicable-disease services that could reduce the burden of multiple visits. Some participants did not recognize weight gain or did not report it when it was not perceived to interfere with daily life; after ART regimen changes, limited communication about possible weight-related effects could also hinder recognition and management (15, 19).

## 4 Discussion

### 4.1 Recognizing the multiple meanings of weight change and aligning patient and provider perspectives

The synthesis showed that the umbrella term “weight change” encompassed clinically distinct phenomena that nevertheless shared overlapping experiential meanings. Illness-associated or unintentional weight loss was commonly linked to worsening health, a poor treatment response, or the risk that HIV status might be inferred from physical appearance. By contrast, restoration of weight lost during illness was often interpreted as evidence of recovery, successful ART, and a return to normal life. These views are rooted in the historical association between HIV and visible wasting. Similar interpretations have been reported among healthcare professionals, who may also regard weight gain as a sign of treatment success (21). However, recovery of weight lost during illness is not equivalent to persistent or excessive gain, which may increase the risk of obesity and metabolic complications (22). In an international cohort of individuals who initiated ART soon after HIV seroconversion, weight increased across different ART classes, suggesting that some early weight gain may reflect a return-to-health effect rather than the influence of a single antiretroviral agent (23). The qualitative findings therefore support distinguishing illness-related weight loss, early weight restoration, and persistent or unwanted gain when discussing patients’ experiences.

Perceptions of weight change were also shaped by stigma and social circumstances. External evidence shows that HIV-related stigma is associated with poorer quality of life (24), and people living with both HIV and obesity may experience enacted and internalized weight stigma (25). Illness beliefs, discrimination, and access to social support may further influence these experiences (26). Evidence linking HIV-status nondisclosure and financial hardship with psychological distress does not address weight change directly, but it illustrates why bodily changes should be discussed within the wider context of patients’ lives (27). The qualitative synthesis suggests that follow-up conversations should explore what weight change means to the individual, which body size they consider acceptable, whether changes in appearance raise concerns about unintended disclosure, and whether they distinguish early weight restoration from persistent or excessive gain. These implications arise from participants’ accounts; they are intended to support patient-centered communication rather than to define clinical thresholds for intervention.

### 4.2 Understanding the multifactorial determinants of weight change and improving clinical assessment and treatment communication

The present synthesis captures participants’ perceptions, beliefs, and causal attributions regarding weight change. It cannot establish that ART, any individual antiretroviral agent, or a specific regimen caused the reported changes in body weight. Participants attributed weight change to ART, appetite, eating patterns, emotional well-being, and medication-related adverse effects, while some attributed weight gain primarily to excessive food intake or other behavioral factors. Considered separately from these qualitative accounts, external clinical evidence indicates that weight trajectories after ART initiation are multifactorial and may vary with immune recovery, baseline disease severity, sociodemographic characteristics, lifestyle, and regimen composition. Differences in BMI trajectories have been observed across ART regimens in real-world settings (28), while pooled analyses have associated greater weight gain after treatment initiation with lower baseline CD4 counts, higher HIV RNA levels, female sex, and dolutegravir- or tenofovir alafenamide-containing regimens (29). However, metabolic outcomes associated with integrase strand transfer inhibitor use vary across populations, indicating that apparent regimen effects should be interpreted in the context of prior treatment exposure and baseline metabolic risk (30). Switching away from regimens containing integrase strand transfer inhibitors or tenofovir alafenamide may limit further gain but does not consistently reverse weight already gained (31). Body image concerns may also affect ART adherence, particularly when accompanied by depressive symptoms (32).

The qualitative findings suggest that clinical encounters should explicitly elicit patients’ explanations of weight change, body image concerns, emotional responses, and intentions to modify or discontinue ART. Specific recommendations for serial monitoring of weight, BMI, waist circumference, metabolic indicators, and cardiovascular risk, however, are drawn from external clinical guidelines and clinical evidence rather than established by the seven qualitative studies themselves. Current guidance supports routine assessment of weight and metabolic risk in comprehensive HIV care (9). Unintentional weight loss warrants evaluation for disease progression, opportunistic infections, malnutrition, gastrointestinal disorders, and other underlying causes, whereas persistent or excessive gain warrants assessment of glycemic status, lipid levels, blood pressure, cardiovascular risk, and functional impact. When no single cause is evident, clinicians should discuss the likely multifactorial nature of the change rather than dismissing patients’ concerns or automatically attributing it to ART. Shared decision-making may help patients make informed treatment choices and reduce unsupervised or clinically inappropriate regimen changes. The Chinese Guidelines for the Diagnosis and Treatment of HIV/AIDS (2024 edition) similarly emphasize management of non-AIDS-related conditions, lifestyle support, and psychosocial care as part of comprehensive HIV management (33).

### 4.3 Addressing multilevel barriers to weight management and improving individualized support

The qualitative synthesis highlights that recognition of healthy eating and physical activity does not necessarily translate into sustained behavior change. Participants described barriers related to physical health, self-efficacy, family routines, financial resources, work demands, cultural expectations, and access to professional support. These accounts indicate that difficulty with weight management should not be reduced to limited motivation or self-discipline. Consistent with these findings, previous qualitative research has identified physical weakness, comorbidities, work-related stress, limited family support, and insufficient professional guidance as barriers to physical activity among people living with HIV (34). Hoosen et al. (35, 36) similarly reported that family encouragement, flexible arrangements, and regular reminders facilitated healthier behaviors, whereas food insecurity, financial hardship, established routines, and culturally embedded dietary practices made change more difficult. Evidence also shows that lifestyle factors have not been assessed consistently in studies of weight gain among people living with HIV (37).

External interventional evidence, considered separately from the present qualitative synthesis, suggests that brief advice alone may be insufficient for sustained change. Structured interventions with ongoing professional support may improve weight-related behaviors, although current evidence is limited by small samples and short follow-up periods (38, 39). Pharmacological treatment may provide an additional option for selected patients. Once-weekly semaglutide reduced body weight and visceral adiposity in adults with HIV-associated lipohypertrophy, but this specific pattern of fat accumulation is not equivalent to all forms of ART-related weight gain, and the findings should not be generalized to the wider population of people receiving ART (40). The reluctance toward injections, additional tablets, and frequent clinic visits identified in this synthesis adds an acceptability perspective to this clinical evidence but does not establish the effectiveness or indication of any specific pharmacological intervention.

The qualitative findings support adapting weight-management support to patients’ practical circumstances and preferences, including affordability, family context, treatment burden, and access to professional support. Potential service approaches may include affordable dietary guidance, group- or home-based physical activity, flexible follow-up, and family or peer involvement. Specific decisions about obesity treatment, metabolic monitoring, referral, and multidisciplinary care should follow external clinical guidance and individual risk assessment rather than being inferred from the qualitative synthesis alone. For patients with obesity, metabolic abnormalities, functional limitations, or substantial body image concerns, coordinated care integrating HIV, nutritional, psychological, and metabolic expertise may be appropriate. The Chinese Guidelines for the Clinical Management of Obesity (2024 edition) provide one framework for such care, provided that recommendations are adapted to the clinical and social context of long-term HIV treatment (41).

### 4.4 Limitations

This review has several limitations. First, only seven studies were eligible, and they did not investigate an identical form of weight change or ask the same qualitative question. The synthesis should therefore be interpreted as an aggregation of cross-cutting experiential themes rather than a comprehensive representation of every form of weight change among people receiving ART. Evidentiary support was also uneven across categories; this is consistent with the CERQual assessment, in which confidence was high for the first synthesized finding but moderate for the second and third because of concerns including adequacy and uneven support. Second, the evidence was geographically concentrated: five studies were conducted in sub-Saharan Africa and two in the United States, with no included studies from Asia, Europe, or Latin America. Sociocultural meanings of body size, HIV-related stigma, food environments, access to care, and ART practices vary across settings; consequently, transferability to underrepresented regions should be assessed cautiously. China is one example of an unrepresented setting rather than the sole context to which transferability is limited. Third, several primary studies used relatively small, purposively selected samples, and some focused on specific subgroups, including women receiving DTG and people with obesity and/or type 2 diabetes. These sampling features may overrepresent experiences of people with pre-existing or clinically salient weight concerns. Qualitative findings are also shaped by self-reported accounts, interview context, researcher interpretation, and reflexivity, which was incompletely reported in several included studies. Accordingly, the synthesis is intended to identify potentially transferable patterns of experience in comparable contexts, not to estimate prevalence, quantify effect sizes, or support statistical generalization to all people living with HIV. Fourth, restricting eligibility to Chinese- and English-language publications and excluding theses, dissertations, and conference abstracts may have introduced language and publication/gray-literature bias and may have omitted culturally distinct experiences; reference-list screening cannot eliminate this limitation. Finally, several studies provided limited information on ART regimens, treatment duration, baseline weight status, and the temporal course of weight change, restricting more detailed comparisons across treatment contexts.

## 5 Conclusion

Weight change among people living with HIV receiving ART carried intertwined medical, psychological, and sociocultural meanings, but the forms of weight change represented in this review were not clinically equivalent. Illness-associated weight loss could revive fears of disease progression and unintended disclosure of HIV status, whereas restoration of weight lost during illness could be interpreted as recovery and treatment success; persistent or excessive gain could raise concerns about body image, function, and metabolic risk. Participants’ accounts also showed that weight-management efforts were shaped by individual capacity, family and social circumstances, cultural expectations, and access to professional support. This qualitative synthesis describes perceptions, beliefs, and causal attributions and does not establish that ART or any specific antiretroviral regimen caused the reported weight changes. The findings support routine exploration of patients’ meanings, concerns, treatment intentions, and practical barriers, while specific monitoring and treatment decisions should be guided by external clinical evidence, relevant guidelines, and individualized risk assessment.

## Data Availability

The original contributions presented in the study are included in the article/Supplementary material, further inquiries can be directed to the corresponding authors.
