# Supplementary material for: Experiences and perceptions of weight change among people living with HIV receiving antiretroviral therapy: a qualitative systematic review and meta-synthesis
Source: Front Public Health. 2026 Oct 27;14:1947513. doi: 10.3389/fpubh.2026.1947513 (PMC13748448; doi:10.3389/fpubh.2026.1947513)
Supplement: Supplementary file 1 [file Data_Sheet_1.PDF]

Supplementary File 1. Complete search strategies for all databases

Searches were conducted from database inception to June 22, 2026. The complete search syntax used for each database and the number of records retrieved are reported below. Two additional records were identified through reference-list screening and were not included in the database total.

Summary of database searches

Database	Search date	Records retrieved
PubMed	June 22, 2026	398
Web of Science	June 22, 2026	445
ProQuest	June 22, 2026	312
Embase	June 22, 2026	272
CINAHL	June 22, 2026	136
PsycINFO	June 22, 2026	54
Scopus	June 22, 2026	635
CNKI	June 22, 2026	2
Wanfang Data	June 22, 2026	41
VIP Database	June 22, 2026	4
SinoMed	June 22, 2026	1
Total		2,300

1. PubMed

#1 ((((((((("HIV"[Mesh]) OR ("HIV Infections"[Mesh])) OR ("HIV"[Title/Abstract])) OR ("HIV infection*"[Title/Abstract])) OR ("human immunodeficiency virus"[Title/Abstract])) OR ("people living with HIV"[Title/Abstract])) OR ("PLHIV"[Title/Abstract])) OR ("AIDS"[Title/Abstract])) OR ("acquired immune deficiency syndrome"[Title/Abstract])) OR ("acquired immunodeficiency syndrome"[Title/Abstract])

#2 (((((("Antiretroviral Therapy, Highly Active"[Mesh]) OR ("Anti-Retroviral Agents"[Mesh])) OR ("antiretroviral therapy"[Title/Abstract])) OR ("anti-retroviral therapy"[Title/Abstract])) OR ("antiretroviral treatment"[Title/Abstract])) OR ("antiretroviral*"[Title/Abstract])) OR ("ART"[Title/Abstract])

#3 ((((((((((("Weight Gain"[Mesh]) OR ("Weight Loss"[Mesh])) OR ("Body Weight Changes"[Mesh])) OR ("Body Image"[Mesh])) OR ("weight gain"[Title/Abstract])) OR ("weight loss"[Title/Abstract])) OR ("body weight change*"[Title/Abstract])) OR ("body image"[Title/Abstract])) OR ("overweight"[Title/Abstract])) OR ("obes*"[Title/Abstract])) OR ("weight management"[Title/Abstract])) OR ("weight change*"[Title/Abstract])) OR ("body size"[Title/Abstract])

#4 (((((((((("Qualitative Research"[Mesh]) OR ("Interviews as Topic"[Mesh])) OR ("Focus Groups"[Mesh])) OR ("qualitative"[Title/Abstract])) OR ("interview*"[Title/Abstract])) OR ("focus group*"[Title/Abstract])) OR ("thematic analysis"[Title/Abstract])) OR ("content analysis"[Title/Abstract])) OR ("phenomenolog*"[Title/Abstract])) OR ("grounded theory"[Title/Abstract])) OR ("mixed method*"[Title/Abstract])) OR ("experience*"[Title/Abstract]) OR ("perception*"[Title/Abstract])

#5 #1 AND #2 AND #3 AND #4

2. Web of Science

#1 TS=("HIV" OR "HIV infection*" OR "human immunodeficiency virus" OR "people living with HIV" OR PLHIV OR AIDS OR "acquired immune deficiency syndrome" OR "acquired immunodeficiency syndrome")

#2 TS=("antiretroviral therapy" OR "anti-retroviral therapy" OR "antiretroviral treatment" OR antiretroviral* OR ART)

#3 TS=("weight gain" OR "weight loss" OR "body weight change*" OR "weight change*" OR "weight management" OR "body image" OR "body size" OR overweight OR obes*)

#4 TS=(qualitative OR interview* OR "focus group*" OR "thematic analysis" OR "content analysis" OR phenomenolog* OR "grounded theory" OR "mixed method*" OR experience* OR perception*)

#5 #1 AND #2 AND #3 AND #4

3. ProQuest

NOFT("HIV" OR "HIV infection*" OR "human immunodeficiency virus" OR "people living with HIV" OR PLHIV OR AIDS OR "acquired immune deficiency syndrome" OR "acquired immunodeficiency syndrome") AND NOFT("antiretroviral therapy" OR "anti-retroviral therapy" OR "antiretroviral treatment" OR antiretroviral* OR ART) AND NOFT("weight gain" OR "weight loss" OR "body weight change*" OR "weight change*" OR "weight management" OR "body image" OR "body size" OR overweight OR obes*) AND NOFT(qualitative OR interview* OR "focus group*" OR "thematic analysis" OR "content analysis" OR phenomenolog* OR "grounded theory" OR "mixed method*" OR experience* OR perception*)

4. Embase

#1 'human immunodeficiency virus infection'/exp OR 'human immunodeficiency virus'/exp OR 'acquired immune deficiency syndrome'/exp OR hiv:ti,ab,kw OR 'hiv infection*':ti,ab,kw OR 'human immunodeficiency virus':ti,ab,kw OR 'people living with hiv':ti,ab,kw OR plhiv:ti,ab,kw OR aids:ti,ab,kw OR 'acquired immune deficiency syndrome':ti,ab,kw OR 'acquired immunodeficiency syndrome':ti,ab,kw

#2 'highly active antiretroviral therapy'/exp OR 'antiretroviral agent'/exp OR 'antiretroviral therapy'/exp OR 'antiretroviral therapy':ti,ab,kw OR 'anti-retroviral therapy':ti,ab,kw OR 'antiretroviral treatment':ti,ab,kw OR antiretroviral*:ti,ab,kw OR art:ti,ab,kw

#3 'weight gain'/exp OR 'weight loss'/exp OR 'body weight change'/exp OR 'body image'/exp OR 'overweight'/exp OR 'obesity'/exp OR 'weight gain':ti,ab,kw OR 'weight loss':ti,ab,kw OR 'body weight change*':ti,ab,kw OR 'weight change*':ti,ab,kw OR 'weight management':ti,ab,kw OR 'body image':ti,ab,kw OR 'body size':ti,ab,kw OR overweight:ti,ab,kw OR obes*:ti,ab,kw

#4 'qualitative research'/exp OR 'interview'/exp OR 'focus group'/exp OR qualitative:ti,ab,kw OR interview*:ti,ab,kw OR 'focus group*':ti,ab,kw OR 'thematic analysis':ti,ab,kw OR 'content analysis':ti,ab,kw OR phenomenolog*:ti,ab,kw OR 'grounded theory':ti,ab,kw OR 'mixed method*':ti,ab,kw

#5 #1 AND #2 AND #3 AND #4

5. CINAHL

S1: SU ("HIV Infections" OR "Acquired Immunodeficiency Syndrome" OR "Human Immunodeficiency Virus") OR TI ("HIV" OR "HIV infection*" OR "human immunodeficiency virus" OR "people living with HIV" OR PLHIV OR AIDS OR "acquired immune deficiency syndrome" OR "acquired immunodeficiency syndrome") OR AB ("HIV" OR "HIV infection*" OR "human immunodeficiency virus" OR "people living with HIV" OR PLHIV OR AIDS OR "acquired immune deficiency syndrome" OR "acquired immunodeficiency syndrome")
S2: SU ("Antiretroviral Therapy, Highly Active" OR "Anti-HIV Agents" OR "Antiretroviral Agents") OR TI ("antiretroviral therapy" OR "anti-retroviral therapy" OR "antiretroviral treatment" OR antiretroviral* OR ART) OR AB ("antiretroviral therapy" OR "anti-retroviral therapy" OR "antiretroviral treatment" OR antiretroviral* OR ART)
S3: SU ("Weight Gain" OR "Weight Loss" OR "Body Weight Changes" OR "Body Image" OR "Overweight" OR "Obesity" OR "Weight Control") OR TI ("weight gain" OR "weight loss" OR "body weight change*" OR "weight change*" OR "weight management" OR "body image" OR "body size" OR overweight OR obes*) OR AB ("weight gain" OR "weight loss" OR "body weight change*" OR "weight change*" OR "weight management" OR "body image" OR "body size" OR overweight OR obes*)
S4: SU ("Qualitative Research" OR "Interviews" OR "Focus Groups") OR TI (qualitative OR "qualitative study" OR "phenomenological research" OR phenomenolog* OR "grounded theory" OR "thematic analysis" OR "content analysis" OR interview* OR "focus group*" OR "mixed method*" OR experience* OR perception*) OR AB (qualitative OR "qualitative study" OR "phenomenological research" OR phenomenolog* OR "grounded theory" OR "thematic analysis" OR "content analysis" OR interview* OR "focus group*" OR "mixed method*" OR experience* OR perception*)
S5: S1 AND S2 AND S3 AND S4

6. PsycINFO

S1: DE ("HIV" OR "AIDS" OR "HIV Infection" OR "Human Immunodeficiency Virus") OR TI ("HIV" OR "HIV infection*" OR "human immunodeficiency virus" OR "people living with HIV" OR PLHIV OR AIDS OR "acquired immune deficiency syndrome" OR "acquired immunodeficiency syndrome") OR AB ("HIV" OR "HIV infection*" OR "human immunodeficiency virus" OR "people living with HIV" OR PLHIV OR AIDS OR "acquired immune deficiency syndrome" OR "acquired immunodeficiency syndrome")
S2: DE ("Antiretroviral Therapy" OR "Drug Therapy" OR "Antiviral Drugs") OR TI ("antiretroviral therapy" OR "anti-retroviral therapy" OR "antiretroviral treatment" OR antiretroviral* OR ART) OR AB ("antiretroviral therapy" OR "anti-retroviral therapy" OR "antiretroviral treatment" OR antiretroviral* OR ART)
S3: DE ("Weight Gain" OR "Weight Loss" OR "Body Weight" OR "Body Image" OR "Obesity" OR "Overweight") OR TI ("weight gain" OR "weight loss" OR "body weight change*" OR "weight change*" OR "weight management" OR "body image" OR "body size" OR overweight OR obes*) OR AB ("weight gain" OR "weight loss" OR "body weight change*" OR "weight change*" OR "weight management" OR "body image"

OR "body size" OR overweight OR obes*)
S4: DE ("Qualitative Research" OR "Interviews" OR "Focus Groups") OR TI (qualitative OR "qualitative study" OR "phenomenological research" OR phenomenolog* OR "grounded theory" OR "thematic analysis" OR "content analysis" OR interview* OR "focus group*" OR "mixed method*" OR experience* OR perception*) OR AB (qualitative OR "qualitative study" OR "phenomenological research" OR phenomenolog* OR "grounded theory" OR "thematic analysis" OR "content analysis" OR interview* OR "focus group*" OR "mixed method*" OR experience* OR perception*)
S5: S1 AND S2 AND S3 AND S4

7. Scopus

#1 TITLE-ABS-KEY("HIV" OR "HIV infection*" OR "human immunodeficiency virus" OR "people living with HIV" OR PLHIV OR AIDS OR "acquired immune deficiency syndrome" OR "acquired immunodeficiency syndrome")
#2 TITLE-ABS-KEY("antiretroviral therapy" OR "anti-retroviral therapy" OR "antiretroviral treatment" OR antiretroviral* OR ART)
#3 TITLE-ABS-KEY("weight gain" OR "weight loss" OR "body weight change*" OR "weight change*" OR "weight management" OR "body image" OR "body size" OR overweight OR obes*)
#4 TITLE-ABS-KEY(qualitative OR "qualitative study" OR "qualitative research" OR interview* OR "focus group*" OR "thematic analysis" OR "content analysis" OR phenomenolog* OR "grounded theory" OR "mixed method*" OR experience* OR perception*)
#5 #1 AND #2 AND #3 AND #4

8. China National Knowledge Infrastructure (CNKI)

TKA=((HIV+AIDS+ 艾滋病 + 获得性免疫缺陷综合征 + 人类免疫缺陷病毒+PLHIV)*(抗逆转录病毒治疗+抗反转录病毒治疗+抗病毒治疗+ART)*(体重增加+体重减轻+体重变化+体重管理+体型+肥胖+超重)*(质性研究+质性访谈+现象学研究+扎根理论研究+混合研究+体验+经历+感知+看法+观点))

9. Wanfang Data

主题:(HIV OR AIDS OR 艾滋病 OR 获得性免疫缺陷综合征 OR 人类免疫缺陷病毒 OR PLHIV) AND 主题:(抗逆转录病毒治疗 OR 抗反转录病毒治疗 OR 抗病毒治疗 OR ART) AND 主题:(体重增加 OR 体重减轻 OR 体重变化 OR 体重管理 OR 体型 OR 肥胖 OR 超重) AND 主题:(质性研究 OR 质性访谈 OR 现象学研究 OR 扎根理论研究 OR 混合研究 OR 体验 OR 经历 OR 感知 OR 看法 OR 观点)

10. VIP Database

((任意字段=HIV OR 任意字段=AIDS OR 任意字段=艾滋病 OR 任意字段=获得性免疫缺陷综合征 OR 任意字段=人类免疫缺陷病毒 OR 任意字段=PLHIV) AND (任意字段=抗逆转录病毒治疗 OR 任意字段=抗反转录病毒治疗 OR 任意字段=抗病毒治疗 OR 任意字段=ART) AND (任意字段=体重增加 OR 任意字段=体重减轻 OR 任意字段=体重变化 OR 任意字段=体重管理 OR 任意字段=体型 OR 任意字段=肥胖 OR 任意字段=超重) AND (任意字段=质性研究 OR 任意字段=质性访谈 OR 任意字段=现象学研究 OR 任意字段=扎根理论研究 OR

任意字段=混合研究 OR 任意字段=体验 OR 任意字段=经历 OR 任意字段=感知 OR 任意字段=看法 OR 任意字段=观点))

11. SinoMed

(("HIV"[全部字段:智能] OR "AIDS"[全部字段:智能] OR "艾滋病"[全部字段:智能] OR "获得性免疫缺陷综合征"[全部字段:智能] OR "人类免疫缺陷病毒"[全部字段:智能] OR "PLHIV"[全部字段:智能]) AND ("抗逆转录病毒治疗"[全部字段:智能] OR "抗反转录病毒治疗"[全部字段:智能] OR "抗病毒治疗"[全部字段:智能] OR "ART"[全部字段:智能]) AND ("体重增加"[全部字段:智能] OR "体重减轻"[全部字段:智能] OR "体重变化"[全部字段:智能] OR "体重管理"[全部字段:智能] OR "体型"[全部字段:智能] OR "肥胖"[全部字段:智能] OR "超重"[全部字段:智能]) AND ("质性研究"[全部字段:智能] OR "质性访谈"[全部字段:智能] OR "现象学研究"[全部字段:智能] OR "扎根理论研究"[全部字段:智能] OR "混合研究"[全部字段:智能] OR "体验"[全部字段:智能] OR "经历"[全部字段:智能] OR "感知"[全部字段:智能] OR "看法"[全部字段:智能] OR "观点"[全部字段:智能]))
